# Incidence of Retinopathy of Prematurity in Extremely Premature Infants

**DOI:** 10.1155/2014/134347

**Published:** 2014-03-09

**Authors:** Alparslan Şahin, Muhammed Şahin, Fatih Mehmet Türkcü, Abdullah Kürşat Cingü, Harun Yüksel, Yasin Çınar, Şeyhmus Arı, İhsan Çaça

**Affiliations:** Department of Ophthalmology, School of Medicine, Dicle University, Diyarbakır, Turkey

## Abstract

*Purpose*. To investigate the incidence and the severity of retinopathy of prematurity (ROP) in extremely preterm infants born before 28 weeks of gestation in southeastern Turkey. *Methods*. A retrospective chart review was performed for infants born before 28 weeks of gestation. The following data were reviewed: gender, gestational age (GA), birth weight (BW), zone and stage of ROP, presence of plus disease, and treatment for ROP if needed. Infants were divided into 2 groups according to GA as follows: group 1 included infants of GAs 25 weeks and under; group 2 included infants of GAs less than 28 weeks and over 25 weeks. *Results*. The incidence of any ROP in the whole cohort, in group 1, and in group 2, was 66.0%, 95.5%, and 58.6%, respectively. Incidence of any ROP was significantly associated with BW and GA (*P* = 0.014 and *P* = 0.002, resp.). The overall incidence of type 1 ROP was 35.8% (59.1% in group 1 and 29.9% in group 2). Development of type 1 ROP was independently associated with GA. *Conclusion*. Any ROP was significantly associated with BW and GA. Extremely premature infants with lower GA were found to be more likely to develop type 1 ROP. BW cannot predict the development of type 1 ROP.

## 1. Introduction

Retinopathy of prematurity (ROP) is an important cause of vision loss in children, especially in extremely premature infants [1]. The survival rates of extremely premature infants have been increased with the improvement in the neonatal intensive care technologies and increased availability of healthcare services in recent years [2].

Infants with gestational age (GA) less than 28 weeks have particularly more risk for the development of ROP in developed countries [3, 4]. In developing countries, the incidence of ROP is rising with the improvement of the survival rates of extremely premature infants [5, 6]. These infants are more tending to develop severe ROP and require treatment [7].

In the present study, we aimed to determine the incidence and the severity of ROP in extremely premature infants in southeast part of Turkey.

## 2. Materials and Methods

Dicle University Health Research Ethics Committee approved the study. The medical records of the premature infants examined between September 2010 and August 2012 in Retina Department of Dicle University Medical Faculty were retrospectively reviewed. The patients with a GA under 28 weeks were included in the study. The patients who died prior to retinal screening examination and had lack of relevant data in their medical records were excluded.

All patients were first examined using indirect ophthalmoscope at 31 weeks of GA and follow-up examinations were performed twice per week to every 3 weeks, depending on the zone and severity of the disease [8]. The pupil was dilated with 2.5% phenylephrine (Mydfrin, Alcon, USA) and 0.5% tropicamide (Tropamide, Bilim, Turkey). The infants were examined after instillation of a topical anesthesthetic, 0.5% proparacaine (Alcaine, Alcon, USA). A lid speculum and scleral indentator were used to visualize the peripheral retina. All examinations were performed by the same ophthalmologist (A.Ş.) experienced in screening premature infants for ROP.

The following data were reviewed: gender, GA, birth weight (BW), zone and stage of ROP, presence of plus disease, and treatment for ROP if needed. The endpoints of data collection in the study defined as completely vascularization of the retina or ROP progressed to the stage require treatment according to ET-ROP recommendations [9].

The infants were divided into two groups on the basis of their GA. Group 1 included infants of GAs 25 weeks and under; group 2 included infants of GAs less than 28 weeks and over 25 weeks. The incidence of any ROP, type 1 ROP, by means of BW and GA, was also evaluated with respect to gender. The description of GA was used as defined by American Academy of Pediatrics [10]. Infants requiring treatment for ROP were defined as having onset of type 1 ROP as used in ETROP study [9]. Treatments were performed either with laser photocoagulation or intravitreal bevacizumab injection. Even though ETROP study recommended ablative laser photocoagulation in the treatment of prethreshold ROP, we preferred intravitreal bevacizumab (IVB) monotherapy since August 2011.

## 3. Statistical Analysis

Mann-Whitney *U*-test was used to compare continuous variables. A *P* value of less than 0.05 was accepted as significant. A logistic regression analysis was used to evaluate the association of GA and BW with development of any ROP and type 1 ROP.

## 4. Results

From September 2010 to August 2012, the medical records of 526 premature infants were reviewed in the Hospital of Dicle University, and 109 of them were eligible for the study. 22 of the premature infants were ≤25 weeks of GA (group 1) and 87 of them were over 25 weeks of GA (group 2). There were 51 male (46.8%) and 48 (53.2%) female infants. The mean BW in groups 1 and 2 was 825.5 ± 211.4 and 997.4 ± 206.2, respectively (*P* < 0.001). The mean GA in groups 1 and 2 was 24.41 ± 0.8 weeks and 26.46 ± 0.5 weeks, respectively (*P* < 0.001). The GA and the BW of the study population were summarized in Table 1.

The incidence of any ROP was 66.0% (72 of 109 infants). The incidence of any ROP in groups 1 and 2 was 95.5% and 58.6%, respectively (*P* = 0.001, *χ* ^2^ = 11.15), with an odds ratio of 15.54 (95% CI 1.99–120.78).

The overall incidence of type 1 ROP was 35.8% (39 of 109 infants). Type 1 ROP incidence in group 1 and group 2 was 59.1% and 29.9%, respectively (*P* = 0.011, *χ* ^2^ = 6.51), with an odds ratio of 3.38 (95% CI 1.29–8.90).  Table 2 summarizes the incidence of the stages of ROP and type 1 ROP according to groups.

The mean GA of infants who had any ROP was 25.8 ± 1.12 weeks and the mean BW was 924.4 ± 205.0 grams. The mean GA of infants who did not have ROP was 26.4 ± 0.6 weeks (*P* = 0.002), and BW was 1036.2 ± 223.8 grams (*P* = 0.014). We did not find any significant difference with respect to the mean BW and GA between patients with and without type 1 ROP (Table 3).

GA was independently associated with any ROP (*P* = 0.018) and type 1 ROP (*P* = 0.038). However BW was not independently associated with any ROP and type 1 ROP (*P* = 0.178 and *P* = 0.714, resp.).

With respect to gender, the comparisons of the incidence of any ROP, type 1 ROP, the mean BW, and the mean GA were not statistically significant (*P* = 0.75, *P* = 0.76, *P* = 0.22, and *P* = 0.75, resp.).

## 5. Discussion

The survival rate of extremely premature infants continuously increases as a consequence of the advancement of neonatal care [5]. These infants are at a particularly high risk for developing more severe ROP and they usually need treatment [3, 11]. In developing countries, severe ROP incidence is also high in more mature infants [5]. On the other hand, there is another risk for the development of the severe ROP is the increase of the survival rates of the extremely premature infants, which may affect the incidence and the severity of ROP in the developing countries [12].

In the current study, the overall incidence of any ROP was 66.0%, which was lower than the 85% incidence reported in the ETROP study [13]. However, similarly, Isaza and Arora [4] reported the overall incidence of any ROP was 64.7%, and Teed and Saunders [11] reported 71%.

The overall incidence of any ROP was 95.5% in group 1 and 58.6% in group 2. Isaza and Arora found the incidence as 88% versus 48%, respectively. Similarly, Teed and Saunders [11] reported the overall incidence of any ROP in the <25 weeks group and in the 25 to 27 weeks group as 87% versus 62%, respectively.

On the other hand, the overall incidence of type 1 ROP was found to be higher in our study (35.8%) than that of previously reported one. Teed and Saunders [11] reported 13% and Isaza and Arora [4] reported 11.6% of patients had type 1 ROP. Gilbert et al. reported that more mature infants were tending to develop severe ROP in less developed countries compared with developed countries [5]. They concluded that criteria of screening programs should be adjusted according to relevant population. Mutlu et al. reported that frequency of ROP in Turkey was similar to that in the United States; however, according to their results, the rate of severe ROP necessitating treatment seems to be higher in Turkey [14].

In the current study, the mean BWs in group 1 and group 2 were 825.5 ± 211.4 and 997.4 ± 206.2 grams, which were higher compared to the previously reported studies conducted in USA [11] and Canada [4]. The survival rates of the infants in newborn intensive care units in developing countries are lower than those of developed countries. We suggest that higher mean BW in our study, compared to developed countries, is the result of lower survival rates of extremely premature infants with relatively lower BW in Turkey. In Sweden, survival rate of extremely preterm infants born before 27 gestational weeks was 70% [15]. The survival rate of extremely premature infants (27 weeks and under) in our newborn intensive care unit was 39%. The mean BW in surviving and nonsurviving infants in this group was 1036.1 ± 273 gr and 780 ± 154 gr, respectively (unpublished data).

We found that the development of type 1 ROP was associated with lower GA but not associated with BW. Woo et al. suggested that GA is a better predictor of ROP than BW according to their twin-paired study [16]. They concluded that maturity is more important in the pathogenesis of ROP than intrauterine growth. Teed and Saunders reported similar findings in their study in infants with a GA under 25 weeks. They suggested that it was a unique characteristic of ROP in extremely premature infants and proposed that other neonatal risk factors may also have some additional effect in the severity and progression of ROP in such infants [11].

In conclusion, our findings suggested that development of any ROP was associated with both lower BW and lower GA. Furthermore, type 1 ROP development was independently associated with GA. On the other hand, no association was found between type 1 ROP and BW in extremely preterm infants with a GA of less than 28 weeks. Although the incidence of any ROP was similar to that of previous reports, the incidence of type 1 ROP in our study population is higher.These results represent only the experience of single center in Turkey and may not be generalized to other different populations. Larger prospective, cohort studies are needed to clarify the relationship of type 1 ROP with BW and GA in extremely premature infants.

## Figures and Tables

**Table 1:** Baseline characteristics of the study population.

Group	*n*	Gender (M/F)	GA (week)		BW (gram)	
			(Mean ± SD)	Range	(Mean ± SD)	Range
1	22	9/13	24.41 ± 0.8	23–25	825.5 ± 211.4	530–1370
2	87	42/45	26.4 ± 0.5	26–27	997.4 ± 206.2	460–1600
All	109	51/58	26.05 ± 1.0	23–27	963.4 ± 217.4	460–1600

BW: birth weight; GA: gestational age; M: male; F: female.

**Table 2:** Distribution of the patients between the groups according to the stages of ROP.

Group	No ROP	Any ROP	Stage 1	Stage 2	Stage 3	AP-ROP	Type 1 ROP
1	1	21	4	8	5	4	13
2	36	51	14	14	15	8	26
All	37	72	18	22	20	12	39

ROP: retinopathy of prematurity.

**Table 3:** The mean BW and GA in patients with and without type 1 ROP in the groups.

Presence of type 1 ROP	BW (gram)			GA (week)		
	Yes	No	*P* value	Yes	No	*P* value
Group 1	826.7 ± 225.7	823.7 ± 200.1	0.828	24.31 ± 0.8	24.56 ± 0.9	0.307
Group 2	972.5 ± 178.2	1008.5 ± 217.9	0.335	26.42 ± 0.5	26.48 ± 0.5	0.656
All	1014.0 ± 220.8	1007.0 ± 206.4	0.899	26.38 ± 0.5	26.48 ± 0.5	0.429

BW: birth weight; GA: gestational age; ROP: retinopathy of prematurity.
